# Protocol for *in vitro* profiling of key TNF downstream signaling pathways involved in cell death and pro-survival signaling in cancer cells

**DOI:** 10.1016/j.xpro.2026.104779

**Published:** 2026-08-12

**Authors:** Laura Unmuth, Britta Lipinski, Julia Harwardt, Lukas Pekar, Stefan Zielonka

**Affiliations:** 1 Antibody Discovery and Protein Engineering (ADPE), Merck Healthcare KGaA, Darmstadt, Germany; 2 Biomolecular Immunotherapy, Institute for Organic Chemistry and Biochemistry, Technical University of Darmstadt, Darmstadt, Germany

**Keywords:** Flow Cytometry, Immunology, Antibody, Biotechnology and bioengineering

## Abstract

Tumor necrosis factor (TNF) is a key mediator of inflammation, acting as a proinflammatory cytokine with context-dependent dual roles. Here, we describe detailed assays to investigate key downstream signaling pathways of TNF involved in either cell death induction or NF-κB-mediated pro-survival signaling via the TNFR1 axis. We describe steps for target cell and reagent preparation, cell stimulation for cell death induction, and detection of activated intracellular signaling proteins by flow cytometry.

For complete details on the use and execution of this protocol, please refer to Unmuth et al.^1^

## Highlights

• Steps for analyzing activated intracellular signaling proteins by flow cytometry
• Detailed instructions for cell stimulation, preparation, and staining protocols
• Guidance for data acquisition, analysis, and gating strategy

## Abstract (editor highlights)

Publisher’s note: Undertaking any experimental protocol requires adherence to local institutional guidelines for laboratory safety and ethics.

## Graphical abstract

**Figure:**
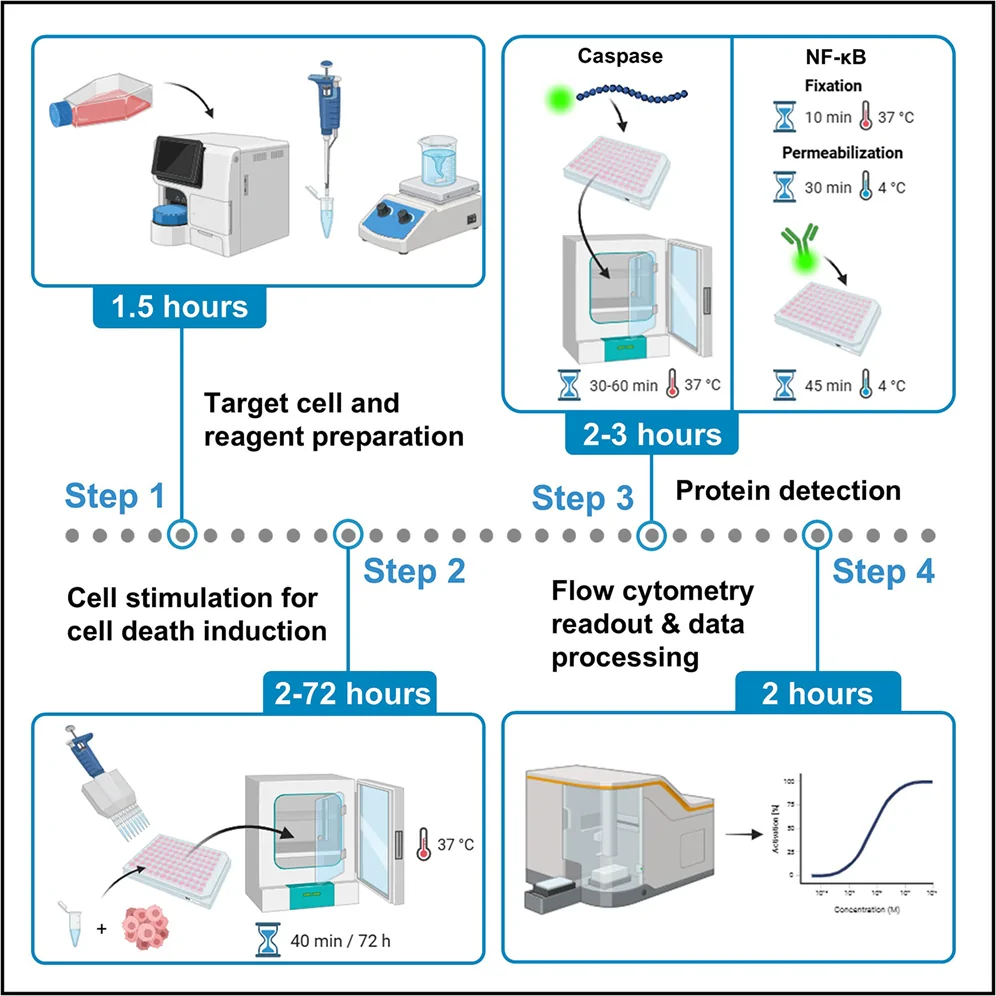

## Before you begin

TNF is a proinflammatory cytokine that modulates immune cell activity and plays a central role in inflammation.^2^ Depending on its form (either membrane-bound or soluble), the cognate TNF receptor (either TNFR1 or TNFR2), as well as the targeted cell type, TNF signaling exhibits pleiotropy, shaping diverse biological responses.^2^^,^^3^^,^^4^^,^^5^ While TNFR1 is widely expressed across most cell types and mainly linked to apoptosis, pyroptosis and necroptosis, TNFR2 is largely restricted to immune cells and associated with cell survival, proliferation and differentiation.^6^^,^^7^^,^^8^ Given its high therapeutic potential, TNF was assessed as recombinant protein for cancer treatment and is approved in Europe for the treatment of patients suffering from metastatic melanoma and soft tissue sarcoma.^9^^,^^10^^,^^11^ However, given its high potency as well as the pleiotropic nature, the therapeutic window of TNF is limited.^12^ To address toxicities associated with TNF therapy, immunocytokines have been developed that are fusion proteins which link a tumor-associated antigen (TAA) targeting paratope to TNF.^12^^,^^13^^,^^14^^,^^15^^,^^16^

Moreover, mutant versions of TNF were developed that display tailored activities towards either TNFR1 or TNFR2.^16^^,^^17^ An alternative to modifying the composition of a cytokine itself for biased activity relies in harnessing bi- and multispecific antibodies which mimic the function of a given cytokine by triggering receptor agonism, referred to as cytokine mimetics.^18^^,^^19^ These antibody-based agonists can be highly engineered to achieve a desired mode-of-action and for multiple different cytokines, cytokine mimetics have been generated.^20^^,^^21^^,^^22^^,^^23^

**Figure 1:**
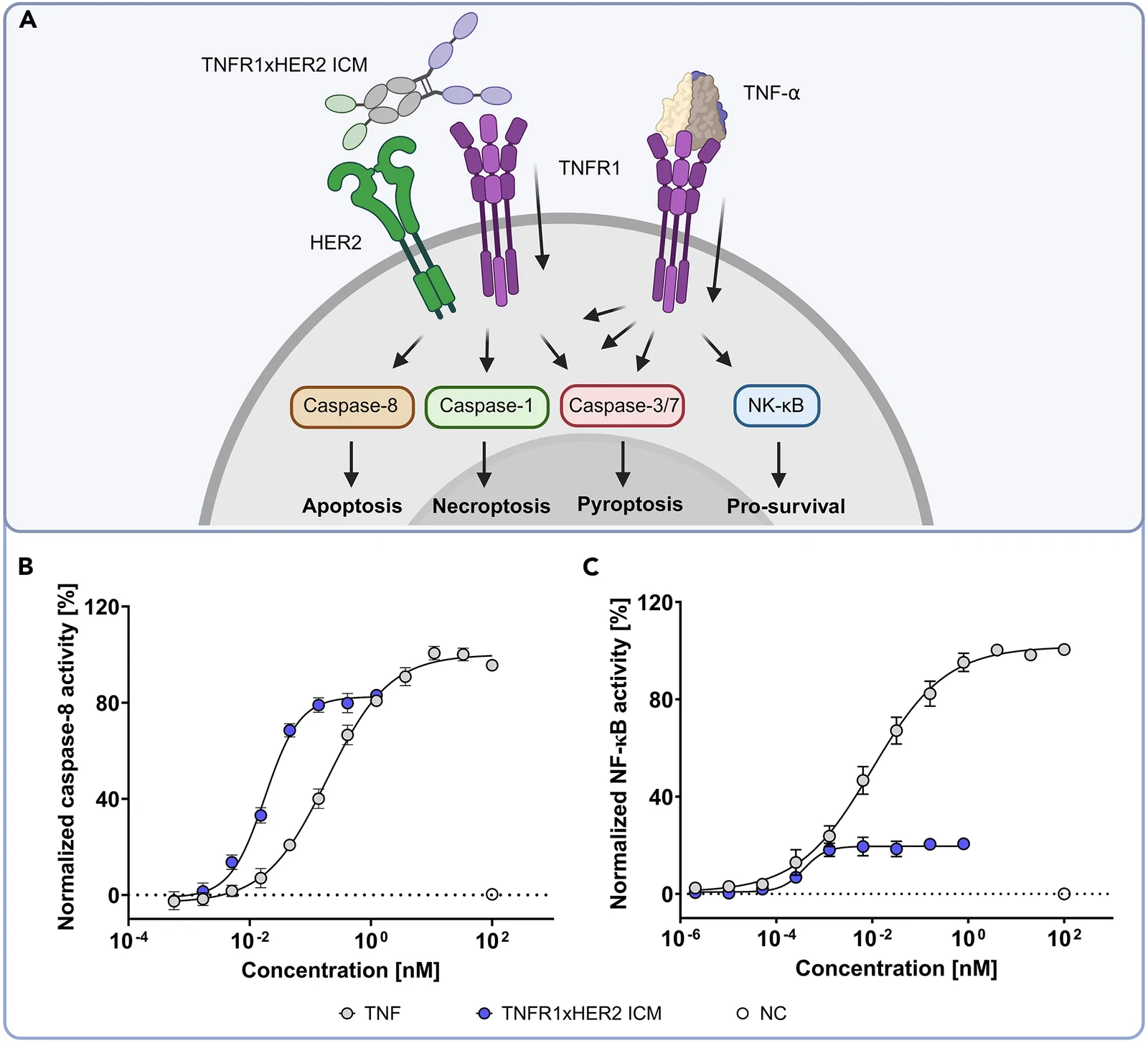
Downstream signaling analysis of induced MCF-7 cells by ICM or TNF to identify the underlying mechanisms of cell death involving caspases and pro-survival signaling (A) Schematic depiction of TNF or TNFR1xHER2 ICM activating the corresponding downstream signaling pathways: caspase-8 inducing apoptosis, caspase-1 inducing necroptosis, caspase 3/7 inducing pyroptosis and NF-κB initiating pro-survival signaling. (B) Strong dose-dependent caspase-8 and weak NK-κB (C) activity in MCF-7 cells after stimulation with TNFR1xHER2 ICM in comparison to TNF. Activity signals were normalized to TNF. Graphs show mean values ± SEM of four independent experiments. Figure was partially created with Biorender.com.

Recently, our group described a strategy for the targeted delivery of TNFR1 agonism to tumor cells in order to preferentially induce cancer cell death via cis delivery.^1^ For this, TNFR1-targeting agonistic single domain antibodies^24^ (sdAbs) were combined in different valencies with HER2-specific sdAbs (Figure 1A).^1^ Resulting bispecifics triggered robust killing of HER2 overexpressing MCF-7 cells as well as caspase induction. Interestingly, this was decoupled from pro-survival signaling via NF-κB.

Here, we detail protocols that enable cell and sample preparation as well as cell stimulation to investigate intracellular signaling pathways involved in cell death induction or pro-survival signaling in cancer cells following TNFR1 stimulation. To this end, cancer cells are stimulated with TNF-like molecules or TNF to detect activated caspase-1, caspase-3, caspase-8 or NF-κB with fluorophore labeled peptides and antibodies by flow cytometry. This protocol is optimized for a fast and miniaturized assay procedure in 96-well scale. As cancer cell line, MCF-7 breast cancer cells were harnessed. Generally, this protocol is also applicable to OVCAR-3 ovarian carcinoma cells or MDA-MB-453 breast cancer cells.

### Pretests for cell line selection and optimal stimulation conditions

Before setting up the experiment the target cell line needs to be tested for its TNF sensitivity to induce cell death in pretests. Cell and compound concentrations as well as incubation time points need to be defined to meet the maximum or appropriate readout range for each cell type.

### Assay volume

The herein presented assay procedures are volume adapted to fit in a 96-well format and to save reagents. The protocol for caspase activity or detection assays was adapted from a total assay volume of 300 μL per sample with 1×10^6^ cells/mL to 100 μL per sample with 0.5×10^6^ cells/mL. Based on this reduction by a factor of three, the added volume of detection reagent was also adapted from 1 μL per sample to 0.3 μL per sample.

The staining volume for NF-κB detection assay was decreased by a factor of eight (from 100 μL to 12.5 μL) was also applied to the detection antibody by adding 2.5 μL instead of 20 μL per sample.

### Innovation

This protocol enables the combined analysis of several mediators of cell death induction as well as pro-survival signaling in cancer cells stimulated with TNF-like molecules. Consequently, it can be used to shed light on underlying mechanisms of cell death such as apoptosis, pyroptosis and necroptosis. Furthermore, it can be harnessed to study pro-survival outcomes via NF-κB activation. By reducing and optimizing assay volumes, the workflow reduces timelines and resources. It can be implemented in a 96-well format to facilitate the simultaneous processing of multiple samples.

### Cell culturing

**Timing: 2 weeks**

Culturing and expanding target cells are mandatory steps prior to starting the experiments.

1. Prepare the following equipment:
  a. Biosafety cabinet.
  b. Pre-heated water bath.
  c. Sterile 50 mL tubes for cell centrifugation.
  d. Centrifuge.
  e. Vacuum aspirator.
  f. Incubator.
  g. Microscope.
  h. Cell culture flask.
2. Prepare complete growth medium/culture media according to the “materials and equipment” section in a sterile environment and store medium at 4°C in a fridge until needed.
3. Prewarm culture media, 1× PBS and Trypsin-EDTA to 37°C in a water bath.
4. Thaw frozen cells in a water bath and resuspend cells in culture media.
  a. Place frozen cell vial (5×10^6^ cells/mL) in a 37°C pre-heated water bath until the cell suspension is completely thawed.
  b. Take up thawed cell suspension in 9 mL culture media in a sterile tube and centrifuge at 500 × *g* for 5 min to remove freezing medium.
  c. Remove the supernatant by aspiration and resuspend cell pellet in 15 mL culture medium for 0.3×10^6^ cells/mL.
  d. Transfer cell suspension into a sterile T75 cell culture flask and incubate at 37°C and 5% CO_2_ in an incubator.
5. Maintain and expand cell culture by passaging and renewing culture media twice a week.
  a. Remove medium from cell culture by gentle aspiration.
  b. Wash cells with 10 mL sterile 1× PBS.
  c. Remove 1× PBS by gentle aspiration.
  d. Add 1 mL trypsin-EDTA directly to the cell monolayer and distribute to completely cover the cells.
  e. Incubate at 37°C for 2 to 3 min.
  f. Check under the microscope if the cells have detached from the plate.
    **CRITICAL:** It is important to ensure that all cells have detached before proceeding to the next step.
  g. Add 9 mL culture media to stop the trypsin-EDTA reaction and transfer cell suspension to a sterile tube.
  h. Centrifuge cell suspension at 500 × *g* for 5 min, aspirate supernatant and take up cell pellet in 10 mL fresh culture media.
  i. Split cell suspension 1:3 in fresh culture media (3.5 mL of cell suspension and 11.5 mL of culture media), transfer into a new sterile T75 cell culture flask and incubate at 37°C and 5% CO_2_ in an incubator.

### Target cell preparation

**Timing: 1 h**

6. Prepare the following equipment:
  a. Biosafety cabinet.
  b. Pre-heated water bath.
  c. Sterile 50 mL tubes for cell centrifugation.
  d. Centrifuge.
  e. Cell counting equipment, such as automated cell counter.
7. Prewarm culture media, 1× PBS and trypsin-EDTA to 37°C in a water bath.
8. Prepare target cell suspension for assay setup:
  a. Remove medium from cell culture by gentle aspiration.
  b. Wash cells with 10 mL sterile 1× PBS.
  c. Remove 1× PBS by gentle aspiration.
  d. Add 1 mL trypsin-EDTA directly to the cell monolayer and distribute to completely cover the cells.
  e. Incubate at 37°C for 2 to 3 min.
    ***Note:*** The incubation duration at 37°C during trypsinization is cell line dependent.
  f. Check under the microscope if the cells have detached from the plate.
    **CRITICAL:** It is important to ensure that all cells have detached before proceeding to the next step.
  g. Add 9 mL culture media to stop the trypsin-EDTA reaction and transfer cell suspension to a sterile tube.
  h. Centrifuge cell suspension at 500 × *g* for 5 min, aspirate supernatant and take up cell pellet in 10 mL fresh culture media.
  i. Count cells with trypan blue in an automated cell counter.
    ***Note:*** Use the cells within the first five to fifteen passages after thawing for any experiments and only if viability is >90% to ensure stable receptor expression and signaling.

### Preparations for caspase detection in flow cytometry

**Timing: 30 min**

9. Prepare the following equipment:
  a. Round-bottom 96-well plate.
  b. Humidity chamber.
  c. Incubator.
  d. Flow cytometer.
10. Thaw commercial activity assay kits and bring all reagents to 20–25°C.
11. Prepare a humidity chamber to prevent medium evaporation during sample incubation.
12. During sample incubation, prepare 1× wash buffer solution according to the “materials and equipment” section and store it at 4°C until needed.
13. Right before staining, prepare 30× FLICA working solution according to the “materials and equipment” section and keep it in the dark until needed.

### Preparations for NF-κB detection in flow cytometry

**Timing: 30 min**

14. Prepare the following equipment:
  a. Round-bottom 96-well plate.
  b. Humidity chamber.
  c. Incubator.
  d. Heating plate shaker.
  e. Ice bucket.
  f. Flow cytometer.
15. At least one day before assay setup, cool down absolute methanol to −20°C.
16. Prepare a humidity chamber to prevent medium evaporation during sample incubation.
17. During sample incubation, prepare washing buffer and BD Phosflow lyse/fix buffer according to the “materials and equipment” section.
  a. Store the washing buffer at 4°C until needed.
  b. Prewarm 1x BD Phosflow lyse/fix buffer to 37°C in a water bath.
18. Pre-warm heating plate shaker to 37°C.
19. Right before staining, prepare detection antibody solution and corresponding isotype control solution according to the “materials and equipment” section and keep in the dark at 4°C until needed.

## Key resources table

**Table:** 

REAGENT or RESOURCE	SOURCE	IDENTIFIER
**Antibodies**		

BD Phosflow™ Alexa Fluor® 488 Mouse anti-NF-κB p65 (pS529), 1:5 diluted in staining buffer	BD Biosciences	Cat#558421; RRID: AB_647091
BD Phosflow™ Alexa Fluor® 488 Mouse IgG2b, κ Isotype Control, 1:5 diluted in staining buffer	BD Biosciences	Cat#558716; RRID: AB_1645613

**Chemicals, peptides, and recombinant proteins**		

Dulbeccos Modified Eagle Medium (DMEM), high glucose, L-glutamine, phenol red	Gibco	Cat#11965092
Fetal bovine serum (FBS)	Gibco	Cat#A5256701
Recombinant human TNF	Acro Biosystems	Cat#TNA-H5228
Phosphate buffered saline (PBS)	Sigma Aldrich	Cat#D8537
Trypsin-EDTA (0.05%)	Gibco	Cat#25300054
Vi-CELL BLU Reagent Kit	Beckman Coulter	Cat#C06019
Bovine serum albumin (BSA)	Sigma Aldrich	Cat#A9418
Methanol	Sigma Aldrich	Cat#322415

**Critical commercial assays**		

BD Phosflow Lyse/Fix Buffer 5×	BD Biosciences	Cat#558049
FAM-FLICA(R) Caspase 1 Assay Kit	Biomol	Cat#ICT-98
CaspaTag™ Caspase-3 In Situ Assay Kit	Merck Millipore	Cat#APT403
CaspaTag™ Caspase-8 In Situ Assay Kit	Merck Millipore	Cat#APT408
PP-conical sterile tube, 50 mL	Falcon	Cat#352070
Vi-CELL sample cups	Beckman Coulter	Cat#383721
T75 cell culture flask adherent	Greiner	Cat#658175
Tissue culture treated 96-well round-bottom plate	ThermoFisher Scientific	Cat#168136
PP 96-deep well round-bottom plate	VWR	Cat#732-3322

**Experimental models: Cell lines**		

Human MCF-7 cells	ATCC	Cat#HTB-22

**Software and algorithms**		

IntelliCyt ForeCyt Software 10.1	Sartorius	N/A
GraphPad Prism version 10.2.1 for Windows	GraphPad Software, Inc.	RRID:SCR_002798

**Other**		

HERAsafe HS12 laminar flow safety cabinet	Heraeus	N/A
Water bath	Memmert	N/A
Refrigerated Centrifuge 5810R	Eppendorf	RRID:SCR_019855
Vacuum aspirator	Vakuubrand	N/A
Vi-CELL BLU Cell Counter	Beckman Coulter	RRID:SCR_026900
Heracell 150 incubator	Heraeus	N/A
Inverted microscope	Leica	N/A
ThermoMixer C 5355 heat/cool shaker mixer with MTP micro plate block	Eppendorf	RRID:SCR_025397
iQue3 flow cytometer	Sartorius	N/A

## Materials and equipment

### Recipes for culture media, buffers, and solutions

#### Culture medium

Prepare complete growth/culture medium by adding 10% (50 mL) of fetal bovine serum (FBS) to 500 mL high glucose DMEM medium under sterile conditions. Store prepared culture medium at 4°C for up to 4 weeks.

***Note:*** Store FBS aliquots at −20°C and thaw in a 37°C water bath prior to use.

#### Wash buffer from caspase assay kits

Prepare 1x wash buffer solution by diluting the 10x concentrate (6 mL) with deionized water (54 mL, for one plate) freshly before each experiment. Store buffer at 4°C until needed.

***Note:*** Before diluting the 10× wash buffer, gently warm the concentrate to dissolve any salt crystals.

#### FLICA working solution for caspase detection

Reconstitute the lyophilized caspase detection reagent (FLICA) with 50 μL DMSO for the 150× stock solution. Mix the reconstituted caspase detection reagent (FLICA) by swirling the vial until completely dissolved and allow the mixture to rest for a few min at 20–25°C. The stock solution is then freshly diluted 1:5 with 1× PBS (60 μL + 240 μL for one plate) to create a 30× working solution. Mix well by vortexing. Store working dilution at 4°C protected from light until needed.

***Note:*** The 150× stock solution can be stored at −20°C for up to 6 months and may be thawed twice during this time. Troubleshooting 1.

**CRITICAL:** Due to the small size (fluorophore-labeled peptide) and the presence of DMSO, there is a risk that the reagent could be rapidly absorbed through the skin upon contact. To prevent skin contact, use gloves, protective clothing and eyewear when handling the FLICA reagent.

#### BD Phosflow lyse/fix buffer (5×)

Prepare 1x BD Phosflow lyse/fix buffer by diluting 1:5 with deionized water (5 mL + 20 mL for one plate) freshly. Buffer should be consumed within one business day.

**CRITICAL:** BD Phosflow lyse/fix buffer is toxic, corrosive and classified as a CMR reagent. Wear gloves, protective clothing and eyewear to avoid skin contact. Dispose the solution and each material that was in contact with the solution in the designated hazardous waste container.

***Alternatives:*** Self-prepared formaldehyde solutions (4% w/v in 1× PBS) can also be used for fixation of cells.

#### Washing buffer for NF-κB detection assay

For one plate, weigh 0.75 g of BSA in a glass bottle, add 150 mL of 1× PBS and stir for about 15 min until BSA is completely dissolved (0.5% w/v). Store at 4°C in a fridge until needed and consume within one business day.

***Alternatives:*** Each commercially available flow cytometry staining buffer containing BSA can be also used, e.g. BD Pharmingen™ Stain Buffer including BSA (BD Biosciences, 554657)

#### Detection antibody solution

Prepare right before staining Alexa Fluor® 488 Mouse anti-NF-κB p65 (pS529) detection antibody dilution and corresponding Alexa Fluor® 488 Mouse IgG2b κ isotype control solution by diluting antibodies 1:5 with washing buffer (for one well: 2.5 μL + 10 μL). Protect from light.

***Alternatives:*** Same antibody clone from another supplier should also work, e.g. anti-human NF-κB p65 pS529 FITC Antibody (Miltenyi, 130-123-548) as well as same clone with another labeled fluorophore, e.g. PE Mouse anti-NF-κB p65 (pS529) (BD Biosciences, 558423)

## Step-by-step method details

### Cell stimulation for cell death induction

**Timing: 2 h up to 3 days**

To analyze the mediators of cell death induction as well as pro-survival signaling during screening of potential TNF-like drug molecules, target cancer cells need to be stimulated for cell death induction. Based on molecule design and cell type, pretests need to be conducted to find the optimal stimulation conditions regarding cell amount, sample concentration and incubation times. This section describes an example protocol for stimulation of MCF-7 cells to induce cell death.

1. Seeding of target cells in assay plate.
  a. Dilute target cell suspension based on cell count to 1×10^6^ cells/mL in culture media.
  b. Seed 50 μL per well into a round-bottom 96-well plate to reach 5×10^6^ cells/well.

Incubate the cells in a humidity chamber at 37°C to allow the cells to grow adherent.

***Note:*** Cell densities need to be tested and optimized for each cell line. Incubation time to allow cells to grow adherent needs to be adjusted for each cell line.

2. Sample preparation and addition.
  a. Prepare sample dilutions with TNF and potential therapeutic drugs in a separate 96-deep well dilution plate starting at 200 nM in a 1:3 serial dilution up to 56 pM in growth medium (serial dilution example: 70 μL + 140 μL).
    ***Note:*** Sample concentrations need to be tested and adjusted to each cell line used.
  b. Add 50 μL of each sample dilution to the seeded target cells to reach a final target concentration of 100 nM.
    ***Note:*** Include also a medium only control as blank.
3. Sample incubation and cell stimulation.
  a. For caspase detection assay, incubate culture plate for 72 h at 37°C and 5% CO_2_ in a prepared humidity chamber.
  b. For NK-κB detection assay, incubate culture plate for 40 min at 37°C and 5% CO_2_ in a prepared humidity chamber.

***Note:*** The optimal incubation time for signaling protein detection could be cell line and compound dependent and should be evaluated for each assay setup and each cell line to reach the highest activity signals in a kinetic experiment.

### Caspase activity assay to identify underlying cell death mechanisms

**Timing: 2 h**

**Figure 2:**
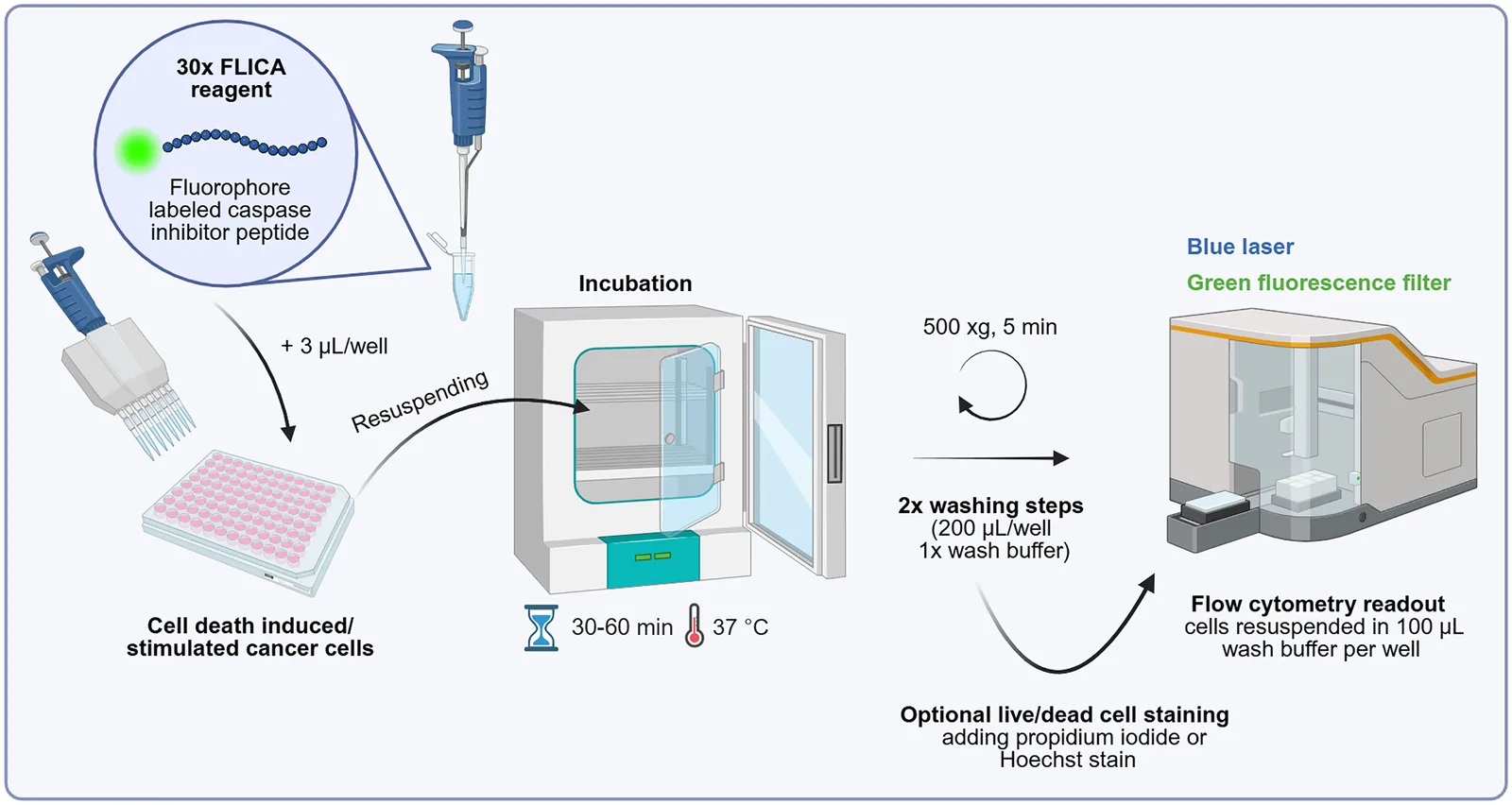
Protocol steps for intracellular detection of activated caspases-1, -3, and -8 with fluorophore labeled caspase inhibitor peptide reagents (1) Prepare 30x FLICA reagent working solution. (2) Add 3 μL 30x FLICA reagent to each well of cell death induced/stimulated cancer cells (100 μL). (3) Resuspend by pipetting with a multichannel pipette or shaking on a plate shaker at 1000 rpm. (4) Incubate at 37°C for 30 min to 1 h. (5) Centrifuge plate at 500 x g for 5 min to remove supernatant, following two washing steps with intermediate centrifuging steps (200 μL/well). (6) Readout plate on a flow cytometer with blue laser und and green fluorescence filter settings after resuspending cells in 100 μL wash buffer per well. (Optional) A live/dead cell staining by adding propidium iodide or Hoechst stain could be included. Figure was created with Biorender.com.

Caspase activation assays can be used to study key downstream signaling pathways involved in cell death induction (Figure 1A). There are several commercial caspase activity assay kits that all use the same principle: specific peptide inhibitors for each caspase are labeled with a fluorophore that exclusively bind to its corresponding caspase and pass through the cell membrane due to its small size without the need of permeabilization. Caspase positive labeled cells can then be analyzed in a flow cytometry measurement. This section describes volume adapted assay steps for the caspase activity kits to scrutinize cells in a 96-well plate format (Figure 2; Table1).

4. After sample incubation (see step 3), add 3 μL of freshly prepared 30x FLICA reagent to each well of the culture plate and incubate for 30 min to 1 h at 37°C and 5% CO_2_ in a humidity chamber protected from light.

***Note:*** After adding the 30× FLICA reagent mix well by resuspending the wells with a multichannel pipette or shaking the plate on a plate shaker at 1000 rpm. Troubleshooting 2.

5. Centrifuge culture plate at 500 x g for 5 min to settle down dead cells in suspension and remove supernatant.
6. Wash cells twice with 200 μL per well of wash buffer with centrifugation steps in between (500 × *g* for 5 min).
7. Resuspend cells in a final volume of 100 μL wash buffer per well.

***Note:*** Additionally, to the detection of caspase activity a live/dead cell staining can be included by adding propidium iodide or Hoechst stain to the final resuspension with wash buffer to quantify death cells (propidium iodide and Hoechst stain solution are included in the kits).

8. Readout plates on a flow cytometer with a blue laser and a green filter setting.

***Note:*** According to the kit instructions it is also possible to perform the readout by fluorescence microscopy or with a fluorescence plate reader.

**Table 1:** Caspase-8 and NF-κB activity of TNFR1×HER2 ICM and TNF in MCF-7 cells as obtained in the original study by Unmuth el al.

Samples	caspase-8 activation		NF-κB activation	
	EC_50_ [pM]	E_max_ [% to TNF]	EC_50_ [pM]	E_max_ [% to TNF]
TNFR1xHER2 ICM	17.8	82.5	0.4	19.6
TNF	191.0	100	9.9	100

### NF-κB detection assay by flow cytometry

**Timing: 3 h**

**Figure 3:**
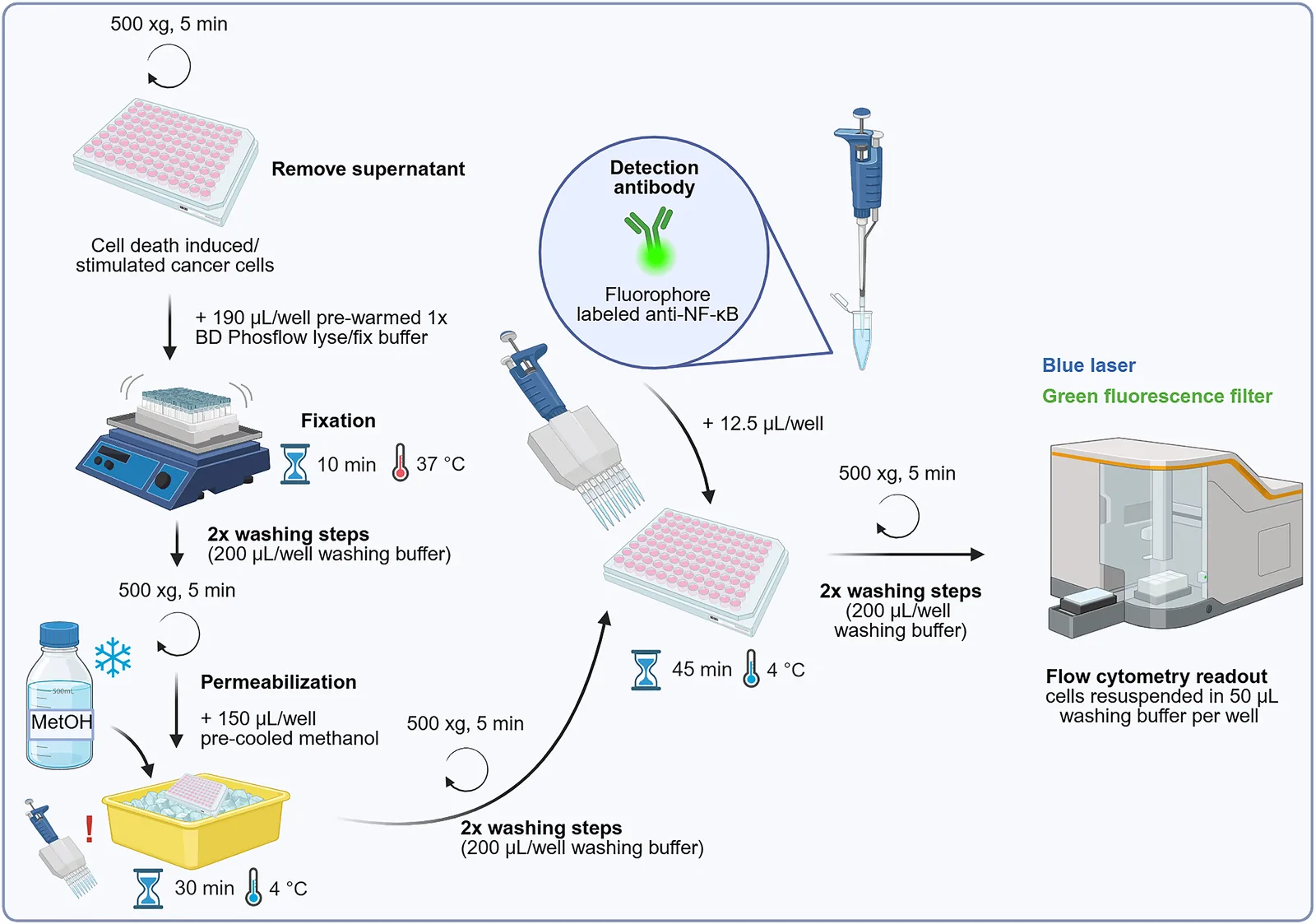
Protocol steps for intracellular staining of activated NF-κB with fluorophore labeled detection antibody (1) Remove supernatant by centrifugation after cell stimulation. (2) Add 190 μL pre-warmed 1× BD Phosflow lyse/fix buffer for cell fixation (37°C, 10 min). (3) Two washing steps with centrifugation steps in between (200 μL/well). (4) Resuspend cells with 150 μL pre-cooled methanol for permeabilization (30 min on ice). (5) Two washing steps with intermediate centrifugation steps (200 μL/well). (6) Add 12.5 μL prepared fluorophore labeled detection antibody to each well and incubation for 45 min on ice. (7) Two final washing steps with centrifugation steps in between. (8) Readout plate on a flow cytometer with blue laser und and green fluorescence filter settings after resuspending cells in 50 μL washing buffer per well. Figure was created with Biorender.com.

One of the major outcomes of TNFR1 agonism is the induction of cell death, while agonism of TNFR2 has been described to trigger cell proliferation and survival.^6^^,^^7^^,^^8^ However, TNFR1 agonism can also trigger pro-survival outcomes via the NF-κB signaling pathway depending on the cell type and the involvement of additional regulatory proteins (Figure 1A).^5^ Consequently, binding of TNF to TNFR1 not only induces apoptosis, pyroptosis and necroptosis, it can also trigger tumor cell growth and survival. This section describes a flow cytometry-based protocol for quantifying intracellular NF-κB levels to assess whether TNF or the tested TNF-like test articles induce pro-survival signaling by triggering TNFR1 agonism. (Figure 3; Table 1).

9. After sample incubation (see step 3), centrifuge culture plate at 500 × *g* for 5 min to settle down dead cells in suspension and decant supernatant.
10. Fixation of the target cells.
  a. Add 190 μL of prepared and pre-warmed 1× BD Phosflow lyse/fix buffer to each well of the culture plate.
  b. Incubate for 10 min at 37°C and 250 rpm on a pre-heated plate shaker. Troubleshooting 3.
11. Centrifuge culture plate at 500 × *g* for 5 min and decant supernatant.
12. Wash cells twice with 200 μL washing buffer per well with centrifugation steps in between (500 × *g* for 5 min).
13. Permeabilize cells with 150 μL pre-cooled absolute methanol for 30 min on ice. Resuspend cells with a multichannel pipette.

**CRITICAL:** For successful cell permeabilization a complete and effective resuspension of cells is mandatory. It is recommended to resuspend cells thoroughly manually several times with a multichannel pipette. Troubleshooting 4.

14. Centrifuge culture plate at 500 × *g* for 5 min and decant supernatant.
15. Wash cells twice with 200 μL washing buffer per well with centrifugation steps in between (500 × *g* for 5 min).
16. Stain the cells with 12.5 μL of prepared anti-NF-κB AF488 labeled detection solution or appropriate AF488 labeled antibody isotype control solution for 45 min on ice in dark.

***Note:*** Cells for detection antibody staining can be resuspended by shaking on a plate shaker instead of pipetting to avoid air bubbles.

17. Wash cells twice with 200 μL washing buffer per well with centrifugation steps in between (500 × *g* for 5 min).
18. Finally, resuspend cells in 50 μL of washing buffer per well.
19. Readout plates on a flow cytometer with a blue laser and a green filter setting.

### Flow cytometry readout data processing

**Timing: 2 h**

This section outlines the data analysis workflow for flow cytometry, detailing a gating strategy to identify target cell population and caspase/NF-κB positive cells as well as calculations to further process, analyze and visualize the data.

20. For data analysis perform a gating strategy with the flow cytometer software (Figure 4) or export FCS files and use another analyzing software, e.g., Flowjo (BD Biosciences):
  a. Draw a gate around the cell population in an FSC-H × SSC-H dot plot.
  b. Perform single cell gating by first excluding cell duplets in an FSC-H × FSC-A dot plot and then in an SSC-H × SSC-A dot plot by drawing a rectangular gate in a 45 degree angle around the main population.
  c. Create a histogram plot with green filter channel as X-axis and place a gate around the desired caspase/NF-κB positive cells so that the medium only control is located left to the caspase positive gate.
  d. Calculate the percentage of caspase/NF-κB positive cells (in gate) in comparison to the whole cell population in IntelliCyt ForeCyt software using the metrics calculation “population count ratio” or determine the geometric mean fluorescence intensity (MFI) in another software and calculate the ratio with excel.

**Figure 4:**
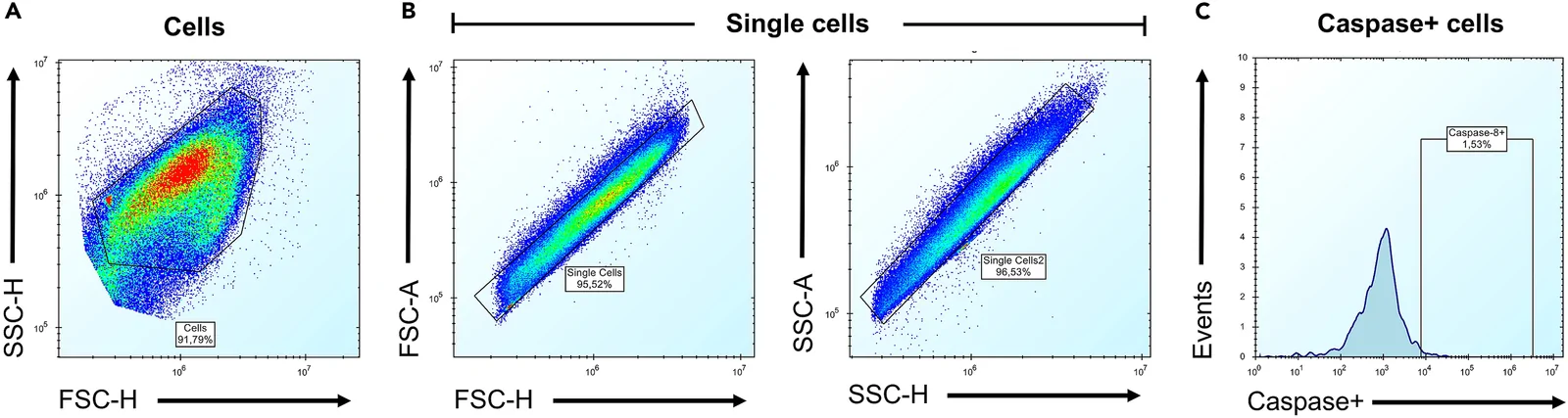
Flow cytometry gating strategy to analyze cells with activated signaling proteins as demonstrated for caspase assay as illustrative example (A) Gating main cell population in an FSC-H × SSC-H dot plot. (B) Double single cell gating by a first FSC-H × FSC-A dot plot followed by an SSC-H × SSC-A dot plot. (C) Histogram plot with set gate around the caspase-8 positive cells based on the negative control. Figure was partially created with Biorender.com.
21. Export the data for further analyses, e.g., to an excel sheet.

## Expected outcomes

Studying key downstream signaling mediators of TNFR1 can provide mechanistic insights into functional properties of potential drug candidates, such as mutant versions of proteins or antibody-based agonists. Our outlined protocol enables the detection of activated signaling proteins that are involved in cell death mechanisms (caspase-1, caspase-3 & caspase-8) as well as cell proliferation and survival (NF-κB) by flow cytometry. As such, researchers can shed light on the different modes-of-action of highly tailor-made TNFR1 agonists to better understand fundamental signaling outcomes after stimulation.

For instance, our group recently generated HER2-directed sdAb-based TNFR1-agonists which, when compared with TNF, decouple cell death induction from pro-survival signaling (Figure 1).^1^ By quantifying the proportion of cancer cells exhibiting activated caspases or NF-κB, this protocol provides insights into the functional ramifications of a given test compound, facilitating the design of molecules with a targeted mode of action.

While this protocol was initially developed harnessing MCF-7 cells, it should be readily amenable for other cancer cell lines, such as OVCAR-3 and MDA-MB-453. Moreover, the assays can be processed in higher throughput once established for a specific cancer cell line to run screenings with multiple potential drug candidates in parallel.

## Quantification and statistical analysis

Further analysis of generated data enables the direct comparison of several potential therapeutic drugs and TNF. Evaluated data can be used to generate dose response curves and calculate EC_50_ as well as E_max_ values. For this, normalize exported data of samples from flow cytometry readout to the highest value of TNF in percentage. Then, plot normalized values in percentage vs molar concentrations with a software (e.g., GraphPad Prism) and fit the dose-response curves with a nonlinear regression curve with four parameters to calculate E_max_ and EC_50_ values.

## Limitations

This protocol enables a fast study of activated signal transduction pathways by flow cytometry and straightforward assay procedure steps, allowing analysis of multiple involved signaling proteins in TNFR agonism and comparison of several potential therapeutic drugs. However, several pretests regarding optimal stimulation conditions and readout time points need to be performed for each target cell line before following the protocol described here. As the activated status of signaling proteins is transient, timing of measurement greatly affects results and bears the risk of missing maximum signal peaks. Moreover, the gating strategy is subjective and therefore not a fixed parameter that could lead to variations. For each cell line and even for the same cell line between several runs, the gating strategy need to be adapted based on the included controls and therefore good prior knowledge in flow cytometry is required. Additionally, variability of labeled detection peptides or antibodies could also affect signal intensity and comparability between different signaling proteins and experiments. Last, the herein presented in vitro protocol may not translate to in vivo tumor biology or patient context.

## Troubleshooting

### Problem 1

No or very low caspase activity signal in flow cytometry (materials and equipment section).

### Potential solution

The reconstituted 150× FLICA stock solution was not stored appropriately or/and thawed more than twice or/and not protected from light.

Immediately after reconstitution, aliquot the 150× stock solution in suitable volumes and store at −20°C in a freezer in the dark when not needed directly for assay preparation.

### Problem 2

Flow cytometry readout analysis shows inhomogeneous assay results.

### Potential solution

Make sure to completely dissolve added caspase detection reagent in the culture medium to ensure uniform staining of the cells by pipetting manually several times up and down with a multichannel pipette or by shaking the plate for 30 s up to 1 min on a plate shaker.

### Problem 3

Cells are not fixed that lead to loss of optimal assay readout point (related to step 10b).

### Potential solution

Prepare pre-warmed lyse buffer for optimal fixation conditions and use a heating plate shaker for a better and direct heat transfer to the culture plates. Incubating culture plates in a 37°C incubator could lead to a worse heat transfer and therefore to an insufficient cell fixation.

### Problem 4

Permeabilization issues decreases staining efficiency (related to step 13).

### Potential solution

Pay attention to resuspend cells with pre-cooled methanol thoroughly to ensure that each cell is surrounded by an adequate amount of methanol and to support cell permeabilization. We advise pipetting manually with a multichannel pipette several times up and down. Take the pre-cooled methanol from the freezer just before use to prevent warming.

## Resource availability

### Lead contact

Further information and requests for resources and reagents should be directed to and will be fulfilled by the lead contact, Stefan Zielonka (stefan.zielonka@tu-darmstadt.de).

### Technical contact

Technical questions on executing this protocol should be directed to and will be answered by the technical contact, Laura Unmuth (laura.unmuth@merckgroup.com).

### Materials availability

Requests for new materials generated in this paper are to be directed to and will be fulfilled (pending MTA and associated restrictions) by the lead contact.

### Data and code availability

• Data reported in this paper will be shared by the lead contact upon request.
• This paper does not report original code.
• Any additional information required to reanalyze the data reported in this paper is available from the lead contact upon request.

## Acknowledgments

We thank Deniz Demir, Ramona Gaa, Hannah Melina Mayer, and Kerstin Hallstein for experimental support. The graphical abstract/figures were created using Biorender.com.

## Author contributions

L.U. developed the protocol. L.U. and B.L. performed all the experiments. L.U., B.L., J.H., L.P., and S.Z. provided analysis and advice. L.U., L.P., and S.Z. conceptually planned and supervised the study. L.U. and S.Z. wrote the manuscript with contributions from all authors.

## Declaration of interests

L.U., B.L., J.H., L.P., and S.Z. are affiliated with Merck Healthcare KGaA. Besides, this work was conducted in the absence of any further commercial interest.
